# Kinetic and thermodynamic properties of purified alkaline protease from *Bacillus pumilus* Y7 and non‐covalent immobilization to poly(vinylimidazole)/clay hydrogel

**DOI:** 10.1002/elsc.201900119

**Published:** 2019-10-24

**Authors:** Yonca Avcı Duman, Nalan Tekin

**Affiliations:** ^1^ Faculty of Arts and Sciences Department of Chemistry Kocaeli University İzmit‐Kocaeli Turkey

**Keywords:** alkaline protease, *Bacillus pumilus*, kinetics, non‐covalent immobilization, PVI/SEP hydrogel, thermodynamics

## Abstract

The characterization of the hydrogel was performed using Fourier‐transform infrared spectroscopy, X‐ray diffraction, and scanning electron microscopy. Purified *Bacillus pumilus* Y7‐derived alkaline protease was immobilized in Poly (vinylimidazole)/clay (*PVI/SEP)* hydrogel with 95% yield of immobilization. Immobilization decreased the pH optimum from 9 to 6 for free and immobilized enzyme, respectively. Temperature optimum 3°C decreased for immobilized enzyme. The *K*_m_, *V*_m_, and *k*_cat_ of immobilized enzyme were 4.4, 1.7, and 7.5‐fold increased over its free counterpart. Immobilized protease retained about 65% residual activity for 16^th^ reuse. The immobilized protease endured its 35% residual activity in the material after six cycle's batch applications. The results of thermodynamic analysis for casein hydrolysis showed that the ΔG^≠^ (activation free energy) and ΔG^≠^_E‐T_ (activation free energy of transition state formation) obtained for the immobilized enzyme decreased in comparison to those obtained for the free enzyme. On the other hand, the value of ΔG^≠^_ES_ (free energy of substrate binding) was observed to have increased. These results indicate an increase in the spontaneity of the biochemical reaction post immobilization. Enthalpy value of immobilized enzyme that was 2.2‐fold increased over the free enzyme indicated lower energy for the formation of the transition state, and increased ΔS^≠^ value implied that the immobilized form of the enzyme was more ordered than its free form.

**Abbreviations**
AIBN: azobisisobutyronitrile
FTIR: Fourier‐transform infrared
GA: glutaraldehyde
IE: immobilization efficiency
PVI: polyvinylimidazole
SEM: scanning electron microscopy
SEP: sepiolite
VIM: 1‐vinyl imidazole
XRD: X‐ray diffraction

## 1 INTRODUCTION

Proteolytic enzymes (proteases) are the enzymes that catalyze the reaction of cleavage of peptide bonds present in proteins 1. Proteases are essential for cell growth and are present in all living cells 2. These enzymes constitute the most important class of industrial enzymes and protease production shares, approximately a quarter of the total enzyme production in the industry. Proteases are generally used in detergent, protein, beer, meat, skin, and dairy industries 3. In enzyme classification, proteases have been included in the hydrolysis group of enzymes. Hydrolysis is performed by an enzyme by hydrolyzing the amide bonds present in the polypeptide chains. Proteases also assist in the synthesis of peptide bonds in certain reactions 4, 5, 6.

Most biological processes occur in the presence of biocatalysts. The acquisition of biocatalysts is expensive, thus the overall economy of their use depends on their recovery and stability. In order to achieve efficient recovery and stability of biocatalysts, immobilization has recently emerged as a system that provides suitable results in terms of high product yield in favorable conditions and allowing the reuse of the biocatalysts. Immobilization allows the efficient production of the desired products by overcoming process limitations, allowing the re‐use of the biocatalysts, increasing stability, and eliminating the undesirable separation as well as the requirement for additional purification steps. Enzyme immobilization allows the same enzyme to be used multiple times. At the end of each usage process, it is very easy to remove the enzyme from the reaction medium, eliminating to the possibility of any biological contamination. The immobilization process increases the efficacy and maintenance of the enzyme 7. Stabilization of monomeric enzymes by multipoint interaction or generation of favorable microenvironments to the enzyme is possible. Multimeric enzymes have been stabilized by immobilizing all enzyme subunits, thus preventing subunit dissociation. However, facts and artifacts can promote improvements in the production of a more active enzyme molecule. These improvements in enzyme activity may be considered an exception to the rule that immobilized enzymes will exhibit a lower catalytic performance. The activity of the enzyme immobilized on the porous surface is higher than the aggregated state of the same enzyme as there are diffusion limitations in the aggregate enzyme. However, this increased activity can be considered as artifact. On the other hand, there may be changes in the hydrophobic or hydrophilic environment of the enzyme after immobilization. This change may improve physicochemical properties. This is a fact effect 8.

The speed and efficiency of the immobilization process depend on the type of carrier (support material), the method of immobilization, concentration, pH, temperature, and reaction time. Strong ionic, hydrophilic or hydrophobic, and/or hydrogen bond interactions between the enzyme and the carrier affect the stability of an enzyme since these strong interactions lead to irreversible adsorption of the enzyme on the carrier, resulting in the loss of enzyme activity. Such strong interactions may also cause conformational changes in the tertiary structure of the enzyme, which again leads to the loss in enzyme activity. These effects may be particularly observed in the case of multiple interactions on the surface of solid carriers 9. A variety of support materials are available for industrial applications, for example, alginate, collagen, chitosan, agar‐agarose, polyacrylamide, polyanhydrides, and clays. On the basis of the production type, cost, product specificity, biocompatibility, and usability, the support material must be considered 10. General expectations from an immobilization process are as follows: providing a balanced bio‐hybrid; providing necessary conditions in which the biomolecules do not get denatured during the process; allowing sufficient contact between the enzyme and the substrate; and allowing to estimate the quantity of biomolecules required for the process 11.

**PRACTICAL APPLICATION**

This work is avaliable for industrial applications.

Several immobilization techniques are available for the immobilization of enzymes. However, the most appropriate one should be selected by considering the result that is required to be achieved. 7. The characteristics of the matrix are important for the catalytic performance of the immobilized enzyme system. The ideal characteristics of a support material include inertness toward enzymes, physical resistance to pressure, hydrophilicity, and the convenience of modification, biocompatibility, and resistance to microbial contamination, and availability at low cost 5. Several methods utilizing different supporting materials have been used for the immobilization of enzymes. Carrier‐binding method utilizes water‐insoluble carriers such as polysaccharide derivatives, synthetic polymers, and glass. In the cross‐linking method, bi‐/multi‐functional reagents such as glutaraldehyde (GA), bisdiazobenzidine, and hexamethylene diisocyanate are used. Membrane confinement method involves the formulation of liposomes and microcapsules. Natural polymers such as collagen, alkaline protease, and j‐carrageenan; synthetic polymers such as polyvinyl alcohol and polyvinylimidazole; or clays such as sepiolite, bentonite, and vermiculite, are used in the trapping and/or non‐covalent interrelating, because it has hydroxyl and amino groups, which easily link with enzymes, together with good hydrophilicity and high porosity 6.

Although immobilization has become a very useful technique as it simplifies the reuse and improves enzyme structural rigidity and activity, but it still has some problems. Enzyme activity losses are very possible when enzymes are cross‐linked by performing chemical modification. Entrapment of enzymes may hardly improve enzyme properties and it is hard to be performed at a large scale. In crosslinked enzymes, the reproducibility is chaotic and enzyme activity losses by chemical modification are very much possible. In addition to these problems, immobilization troubles of the protease enzyme are another group apart. In harsh reaction conditions where urea or guanidine is used, protease must be formed very stable. Large protein substrates create the diffusional limitation which causes the decrease in activity and requires support materials with large pores. This lowers the surface area and the mechanical resistance of the support 12.

However, novel support materials providing high activity and stability must be investigated in order to obtain reusability and economic summits. The use of polyvinylimidazole/sepiolite (PVI/SEP) hydrogel as support materials for protease immobilization has not been identified in the literature. Therefore, in the present study, the PVI/SEP hydrogel was synthesized, using which the alkaline protease enzyme obtained from *Bacillus pumilus* Y7 was immobilized. Glutaraldehyde without purification was used to aggregate the alkaline protease enzyme. PVI/SEP was comparatively investigated using scanning electron microscopy (SEM), X‐ray diffraction (XRD), and Fourier‐transform infrared (FTIR) spectroscopy. The immobilization process was optimized, and the properties of the free and immobilized alkaline proteases were systematically investigated. Finally, the catalytic efficiency, reusability, and the kinetic and thermodynamic properties of the immobilized alkaline protease were evaluated, and compared with those of the free alkaline protease.

## 2 MATERIALS AND METHODS

### 2.1 Materials

Azobisisobutyronitrile (AIBN), 1‐vinyl imidazole (VIM), and sepiolite were purchased from Fluka A.G. (Buch, Switzerland), Aldrich Chem. (Steinheim, Germany), and Aktaş Lületaşı (Eskişehir, Turkey), respectively. The chemical composition of sepiolite as determined by X‐ray fluorescence was: 53.47% SiO_2_, 23.55% MgO, 0.71% CaO, 0.43% NiO, 0.19% Al_2_O_3_, and 0.16% Fe_2_O_3_, with 21.49% loss on ignition. The specific surface area of sepiolite was calculated to be 342 m^2^ g^−1^ 13. Alkaline protease used in the present study (E.C.3.2.1.4) was obtained from *Bacillus pumilus Y7* which was isolated from soil in a previous study by our research group 14. All the other chemicals and solvents used in the present study were of analytical grade and were purchased from Sigma (USA), Fluka AG (Switzerland), Riedel AG (Germany), and Merck AG (Germany).

### 2.2 Synthesis of cross‐linked poly (vinylimidazole) hydrogel

In order to obtain cross‐linked PVI, VIM was polymerized using in situ polymerization as described in previous reports 15, 16, 17. First, AIBN crystallized in methanol was dissolved in 10 mL VIM as it is not soluble in water. Following this, 10 mL of deionized water was added to the aforementioned solution, and the resultant suspension was stirred for 10 min. *N*,*N*’‐Methylenebisacrylamide (0.2 g) was added to the mixture to serve as the cross‐linking agent. The resultant mixture was transferred to a glass‐sealed polymerization reactor, which was in turn placed in a water bath. The contents of the reactor were heated to 65°C under a nitrogen atmosphere for 200 min with continuous stirring. After the hydrogel synthesis, the prepared cross‐linked PVI hydrogel was washed three times with deionized water, followed by drying in a vacuum oven. The dried hydrogel was ground into powder form and stored in a desiccator.

### 2.3 Synthesis of poly (PVI/SEP) hydrogel

Initially, the sepiolite sample was dried at 105°C for 24 h, followed by grinding and sieving to obtain a 75‐µm‐sized fraction. The PVI/SEP hydrogel was synthesized using in situ polymerization as described in the previous studies 18. AIBN was dissolved in 10 mL VIM, followed by the addition of 10 mL of deionized water and a certain amount of sepiolite into the solution. In order to achieve satisfactory dispersion of sepiolite, the prepared mixture was stirred thoroughly for 3 h in an ultrasonic bath. *N*,*N*’‐Methylenebisacrylamide (0.2 g) was added to the mixture, and the resultant mixture was subsequently placed in a water bath at 65°C under a nitrogen atmosphere for 200 min. After hydrogel synthesis, the prepared hydrogel was washed with deionized water in order to remove the residual non‐polymerized VIM and other water‐soluble materials. The PVI/SEP hydrogel was dried in a vacuum oven, ground into powder form, and stored in a desiccator.

### 2.4 Characterization of PVI/SEP hydrogel

The prepared PVI/SEP hydrogel was characterized using different analysis techniques, such as XRD, SEM, and FTIR spectroscopy to determine its structural and morphological properties. FTIR spectra of the prepared hydrogel were recorded using a Bruker Tensor 27 spectrophotometer. The spectra were obtained using Diamond‐based attenuated total reflectance in the range of 4000–400 cm^–1^, over 30 scans, with a resolution of 2 cm^–1^. The XRD patterns of the prepared PVI/SEP hydrogel were obtained using an X‐ray diffractometer (Rigaku Ultima IV). The experimental conditions were scanned at a rate of 1°min^−1^, and the copper K alpha (CuKα) radiation was generated at a voltage of 40 kV and a current of 30 mA. Morphologies of the prepared PVI/SEP hydrogel were examined by using QUANTA 400F field emission scanning electron microscope system which was equipped with an energy dispersive X‐ray spectrometer operating at an accelerating voltage of 10 kV. The surfaces of the cross‐linked PVI and the prepared PVI/SEP hydrogel were sputter‐coated with gold for the SEM analysis.

### 2.5 Purification of alkaline protease

All the steps of the purification were performed at 4°C. The crude enzyme solution was fractionated at a flow rate of 3 mL/min by using single step hydrophobic interaction chromatography. NaCl was directly added into the crude extract enzyme solution to reach the final NaCl concentration of 3 M. Subsequently, 20 mL of this crude extract was applied to the Phenyl Sepharose 6 fast flow high sub. (HIC) (2.5 × 10 cm) column (GE Healthcare, Sweden) that had been previously equilibrated with 50 mM K_2_HPO_4_‐KH_2_PO_4_ phosphate buffer (pH 8) containing 3 M NaCl. The elution of the adsorbed protein was achieved by applying a decreasing (3–0 M) NaCl gradient. The purity of each fraction was evaluated using SDS‐PAGE, and the concentration of the collective fraction was determined through the Bradford assay using BSA as standard 19.

### 2.6 Hydrolytic activity of the alkaline protease

Activities of the free and the immobilized alkaline proteases were determined by mixing 0.5 mL enzyme solution with 2.5 mL casein substrate in a reaction mixture containing 50 mM (0.6%) K_2_HPO_4_‐KH_2_PO_4_ phosphate buffer (pH 8). The reactions were allowed to occur at 30°C for 20 min, followed by termination of the reaction by adding 2.5 mL of trichloroacetic acid [0.11 M TCA, 0.22 M sodium acetate, and 0.33 M acetic acid] and incubating the final solution at room temperature for 30 min. Subsequently, the mixture was filtered, and the filtrate was used for activity analysis using BioRad Smart Spec 3000 at 280 nm 20. One unit of alkaline protease activity was defined as the amount of enzyme required for the production of 1 µg of tyrosine in 1 min under the assay conditions. All the activity measurements were conducted five times independently, and the mean values were used as the final activity values. Standard errors calculated for the mean values were lower than 4%.

### 2.7 Immobilization of alkaline protease

#### 2.7.1 To PVI/SEP hydrogel

Alkaline protease immobilization was performed using glutaraldehyde as the aggregating agent for achieving the encapsulation of alkaline protease in the PVI/SEP hydrogel. Approximately 1 g of the PVI/SEP hydrogel was dispersed in 10 mL enzyme extract (K_2_HPO_4_‐KH_2_PO_4_ phosphate buffer [pH 8]; specific activity: 123.02 U/mg) at 4°C, under shaking conditions at 200 rpm for 1 h. Subsequently, 10 mL (6%) glutaraldehyde was added to the mixture, followed by shaking at 4°C and 200 rpm for 1 h. The Immobilized alkaline protease was separated through decantation and was washed three times with the same buffer solution in order to remove the unabsorbed soluble enzyme. At the end of the immobilization process, all the resultant supernatants were collected, and the final volume was used to determine the amount of bound protein. The immobilization efficiency (IE%) was determined indirectly as the difference between the enzyme activity (U) present in the enzyme solution before and after the immobilization. The immobilization efficiency% was calculated from the following formula: Immobilization efficiency% = Activity of immobilized enzyme/Initial activity of soluble enzyme × 100.

#### 2.7.2 To PVI

PVI (1 g) was dispersed in 10 mL enzyme extract (K_2_HPO_4_‐KH_2_PO_4_ phosphate buffer [pH 8]; specific activity: 120.0 U/mg). The alkaline protease solutions were stirred in the presence of glutaraldehyde or without glutaraldehyde in separate flasks for 1 h at 4°C in order to achieve enzyme interrelating in the hydrogel. At the end of the immobilization process, all the supernatants were collected, and the final volume was used to determine the amount of bound protein. The IE% was determined indirectly as the difference between the enzyme activity (U) present in the enzyme solution before and after the immobilization.

#### 2.7.3 To sepiolite

Clay (1 g) was mixed with 10 mL alkaline protease stock solution (specific activity: 120.0 U/mg). After 1 h of stirring at 4°C, the clay–enzyme suspension was centrifuged at 15 000 × g for 10 min at 4°C. The pellet thus obtained was washed five times with the buffer used in the determination of enzyme activity (K_2_HPO_4_‐KH_2_PO_4_ phosphate buffer [pH 8]). The supernatant obtained post centrifugation was tested for enzyme activity. Alkaline protease activity was estimated in all the supernatants, in the pellet, as well as in the stock enzyme solution, and expressed as the IE%; the values were expressed in terms of grams of dry sepiolite.

### 2.8 Reusability of immobilized enzyme

In order to investigate the reusability of the immobilized alkaline protease, the immobilized enzyme was recovered through decantation after each batch, followed by washing several times with K_2_HPO_4_‐KH_2_PO_4_ phosphate buffer (pH 8) in order to prepare the enzyme for the next batch. All experiments were performed in duplicate, and the results represented the mean values of three independent experiments.

### 2.9 Operational stability of immobilized enzyme

The operational stability of the immobilized enzyme was determined through casein hydrolysis at 30°C using 20 mL of the substrate, i.e. 0.6% casein in 50 mM K_2_HPO_4_‐KH_2_PO_4_ buffer (pH 8) and 0.1 g of immobilized alkaline protease. At the end of each reaction (20 min), homogenous samples were collected and subjected to centrifugation (5000 × g, 15 min, room temperature), and the supernatant obtained was utilized for determining the alkaline protease activity. All experiments were performed in duplicate, and the results represented the mean values of three independent experiments

### 2.10 Characterization of immobilized enzyme

The optimal temperature for the PVI/SEP‐immobilized alkaline protease enzyme was determined at pH 8. In order to determine the optimal pH of the enzyme, the enzyme activity was evaluated first at different pH values ranging from 4 to 10. The relative enzyme activity was calculated as a percentage with respect to the activity of the enzyme at the optimum pH and temperature (100%).

### 2.11 Kinetic characterization of the immobilized enzyme

The kinetic study of the alkaline protease enzyme immobilized on PVI/SEP hydrogel was performed using casein as a substrate. The kinetic parameters were determined under the initial reaction conditions according to the Lineweaver–Burk plot using linear regression. The *K*_m_ (Michaelis–Menten constant) value indicates the affinity of enzyme toward the substrate, the *V*_max_ value reflects the maximum velocity of the enzymatic reaction, and the *k*_cat_ value (turnover number) represents the number of substrate molecules turned over per enzyme molecule per minute 21. All experiments were performed in duplicate, and the results represented the mean values of three independent experiments.

### 2.12 Thermodynamic parameters of the immobilized enzyme

The temperature dependence of the rate constant (below the inactivation temperature) of an enzyme‐catalyzed reaction is expressed by the Arrhenius equation, which is given below:

$k=A\;exp(-\;E_{a}/RT),$

where *k* is the rate constant, *A* is the pre‐exponential factor, *E*_a_ is the activation energy, *R* is the gas constant, and *T* is the absolute temperature in Kelvin. The activation energy (*E*_a_) is calculated from a plot of l*n k* against 1/*T*.

The other thermodynamic parameters of alkaline protease [activation free energy (Δ*G* ^≠^), free energy of transition state formation (Δ*G* ^≠^_E−T_), and free energy of substrate binding (Δ*G* ^≠^_ES_) for casein hydrolysis] for the free and immobilized enzymes were calculated using the following equations, which were originally derived from Eyring's transition state theory 21:

$$\Delta G^{\neq }=-RTln\left(k_{cat}h/k_{B}T\right)\;\left(kjmol^{-1}\right)$$

$$\Delta {G^{\neq }}_{ES}=-RTln\left(1/K_{m}\right)\;\left(kJmol^{-1}\right)$$

$$\Delta {G^{\neq }}_{E-T}=-RTln\left(k_{cat}/K_{m}\right)\;\left(kJmol^{-1}\right)$$

where *R* is the gas constant (8.314 J K^−1^ mol^−1^), *h* is the Planck's constant (6.63×10^−34^ J s), and *k*_B_ is the Boltzmann constant (1.38×10^−23^ J K^−1^).

## 3 RESULTS AND DISCUSSION

Imidazole is one of the most important heterocyclic aromatic compounds. The ring present in the imidazole structure plays an essential role in the interaction with primary biomacromolecules, such as proteins, amino acids, nucleic acids, hormones, and certain vitamin. The imidazole ring holds a significant place in several pharmaceutical applications as well. Polyvinylimidazole (PVI) synthetic macromolecules constitute the rising members of the polymer industry. Imidazole‐containing polymers have been used as components of novel amphiphilic co‐networks for catalyst supports and biomaterials exhibiting various pharmacological activities 22.

### 3.1 Characterization of PVI/SEP hydrogel

#### 3.1.1 FTIR analysis

**Figure 1:**
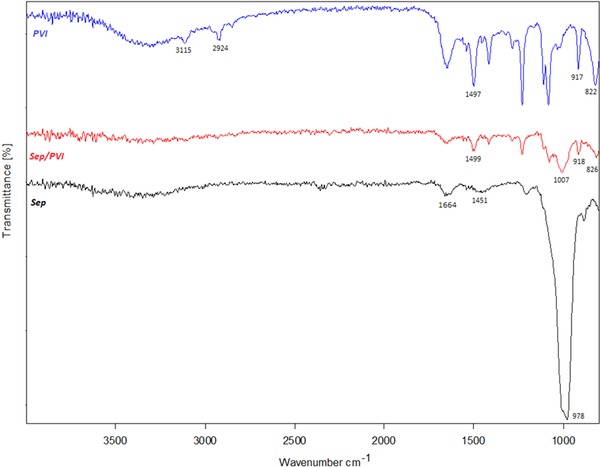
FTIR spectra of sepiolite, PVI, and SEP/PVI hydrogel

The FTIR spectra of sepiolite, PVI, and PVI/SEP are presented in Figure 1. In the infrared spectrum of sepiolite, the absorption peaks were detected between 3000 and 3800 cm^−1^ due to water absorptions and due to the stretching vibrations of the hydroxyl groups present 23. The characteristic band at 1664 cm^−1^ was assigned to the O–H stretching of zeolitic water 24. The peaks at 1451 and 978 cm^−1^ were attributed to O–H bending and Si–O stretching, respectively 24, 25. The peak obtained at 883 cm^−1^ was because of the Si–O–Si symmetric stretching vibrations in the tetrahedral sheet 11. Furthermore, PVI exhibited adsorption bands at 3115, 2924, 1497, 917, and 822 cm^−1^,which were associated with C−H (ring) stretching, C−H (chain) stretching, C−C stretching, and C−N stretching of the imidazole rings, respectively 25. While the FTIR spectra obtained for the PVI/SEP hydrogel were almost similar to those of the pure PVI spectra, the characteristic peak wavenumber of the hydrogel was observed to shift to higher wavenumbers compared to the *PVI* peaks. Nevertheless, anovel band was detected at 1007 cm^–1^, probably due to the Si–O absorption bond of sepiolite with the synthesized hydrogel. A similar result was reported by Tekin et al. in 2016 13. It was determined that the FTIR spectrum of the *PVI/SEP* hydrogel exhibited the characteristic absorption peaks for both sepiolite and PVI, thereby demonstrating successful synthesis of the hydrogel through in situ polymerization.

#### 3.1.2 XRD analysis

**Figure 2:**
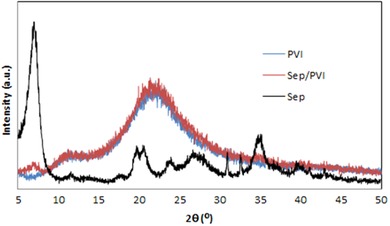
XRD patterns of sepiolite, PVI, and PVI/SEP hydrogel

The XRD patterns for sepiolite, PVI, and PVI/SEP are depicted in Figure 2. In the case of sepiolite, the peak located at 7.01 could be attributed to the reflections of the internal channel 25. The interlayer spacing in PVI/SEP (*d_110_* = 12.72 nm, 2*θ* = 6.95°) was observed to have increased slightly in comparison to that in sepiolite (*d_110_* = 12.60 nm, 2*θ* = 7.01^ο^), while the diffraction peak was observed to have lowered slightly in its intensity. The obtained difference in the interlayer spacing could be related to the insertion of *PVI* between the sepiolite layers. Certain characteristic peaks of sepiolite were observed to disappear in the hydrogel patterns, while the diffraction peak 13 was visible in the pattern of PVI/SEP hydrogel with considerably less intensity, which might be due to individual tetrahedral octahedral tetrahedral (T‐O‐T) layers which bonded strongly through covalent interactions in the sepiolite structure 13, 28. The aforementioned results confirmed that sepiolite was successfully dispersed homogenously in PVI through in situ polymerization 29.

#### 3.1.3 SEM analysis

**Figure 3:**
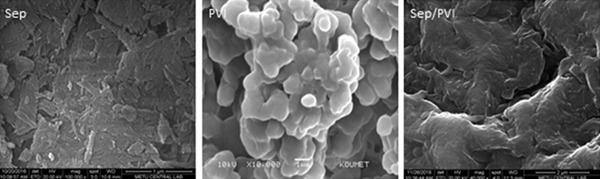
SEM images of sepiolite, PVI, and PVI/SEP hydrogel

The SEM images of sepiolite, PVI, and PVI/SEP hydrogel are depicted in Figure 3. Sepiolite exhibited a fibrous morphology, with an abundance of fibers congregated into bundles. The SEM images of *PVI* depicted micro‐sized particles and a smooth and spherical particulate structure. As seen in the SEM image of the PVI/SEP hydrogel, the surface of sepiolite was covered by PVI, and the PVI/SEP hydrogel was observed to have smooth and dense surfaces. In addition, it was demonstrated that there was a high dispersion of sepiolite in PVI, as confirmed by a strong interfacial adhesion observed between PVI and sepiolite. In the SEM image of the hydrogel, single sepiolite fibers or even bundles were not conveniently observable, which could be attributed to the strong hydrogen bonding between PVI and the silanol groups of sepiolite, and to the high surface area of sepiolite 30, 31.The SEM results were consistent with the XRD results obtained in the present study.

### 3.2 Purification of alkaline protease

**Figure 4:**
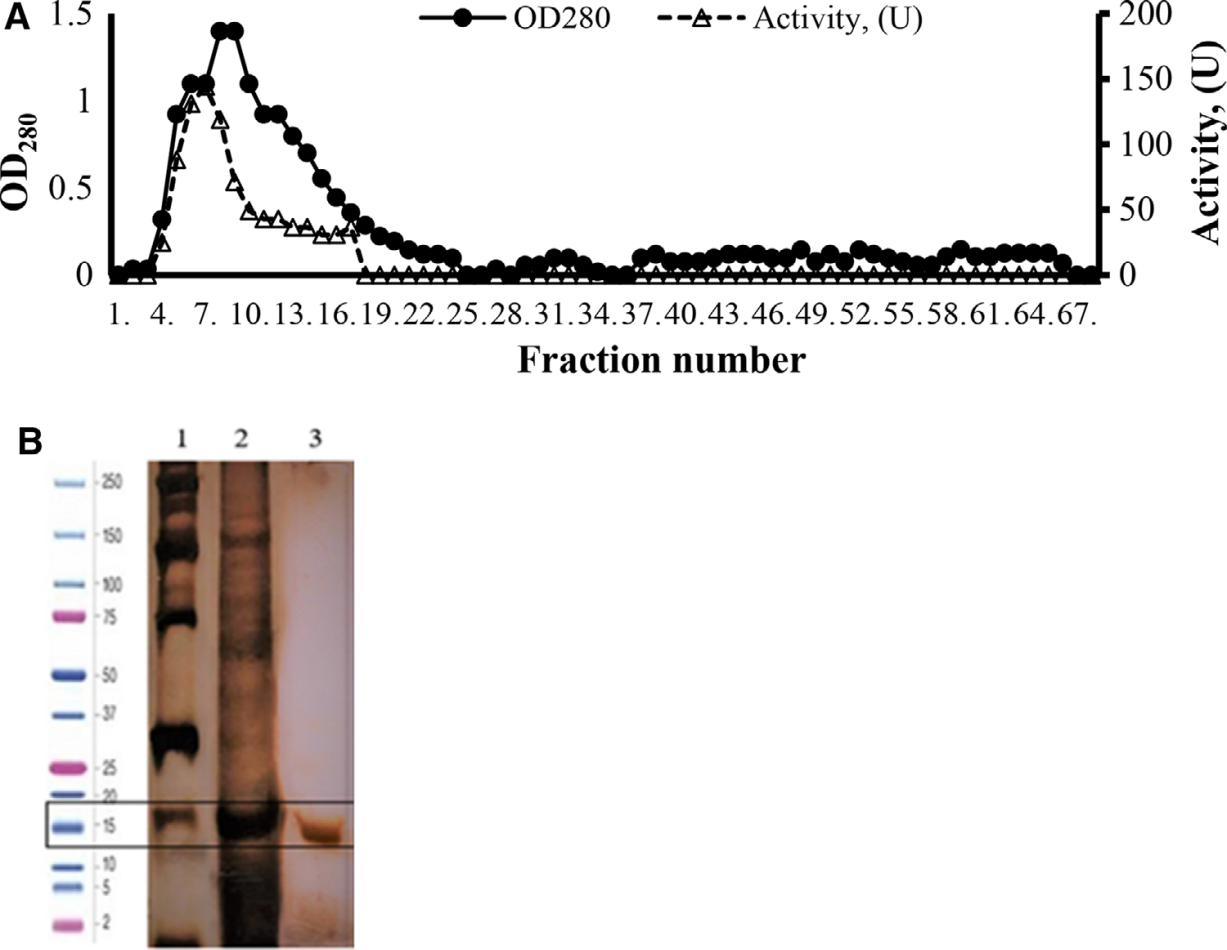
(A) *Bacillus pumilus* Y7 alkaline protease purification by using Phenyl Sepharose high performance column at pH 8.0 and 3 M NaCl. (B) Electrophoretic analysis of the alkaline protease, (1: Molecular weight marker (SeeBlue Plus2 Pre‐stained Protein Standard, LC5925), 2: crude enzyme, 3: purified enzyme)

The alkaline protease enzyme was purified from the culture broth of *B. pumilus* Y7 using single‐step hydrophobic‐interaction chromatography. Utilization of 3 M NaCl and pH 8.0 resulted in the most efficient purification providing selective passage of the protease through the column without binding while the majority of contaminating proteins got adsorbed on the column (Figure 4A). A 5.4‐fold purification with a recovery yield of 52.8% in comparison to the original crude extract was achieved in the process (Table 2).

The molecular weight of the purified enzyme was estimated to be approximately 15 kDa, as confirmed by the presence of a single protein band in the denatured gel. The activity staining process also revealed an activity band of the enzyme corresponding to the size of approximately 15 kDa in the standard lane (Figure 4B). Alkaline proteases usually have molecular weights ranging from 15 to 30 kDa. However, alkaline proteases with molecular weights 31.6, 33, 36, and 45 kDa have also been reported in the literature 32

### 3.3 Immobilization of alkaline protease on hydrogel

**Figure 5:**
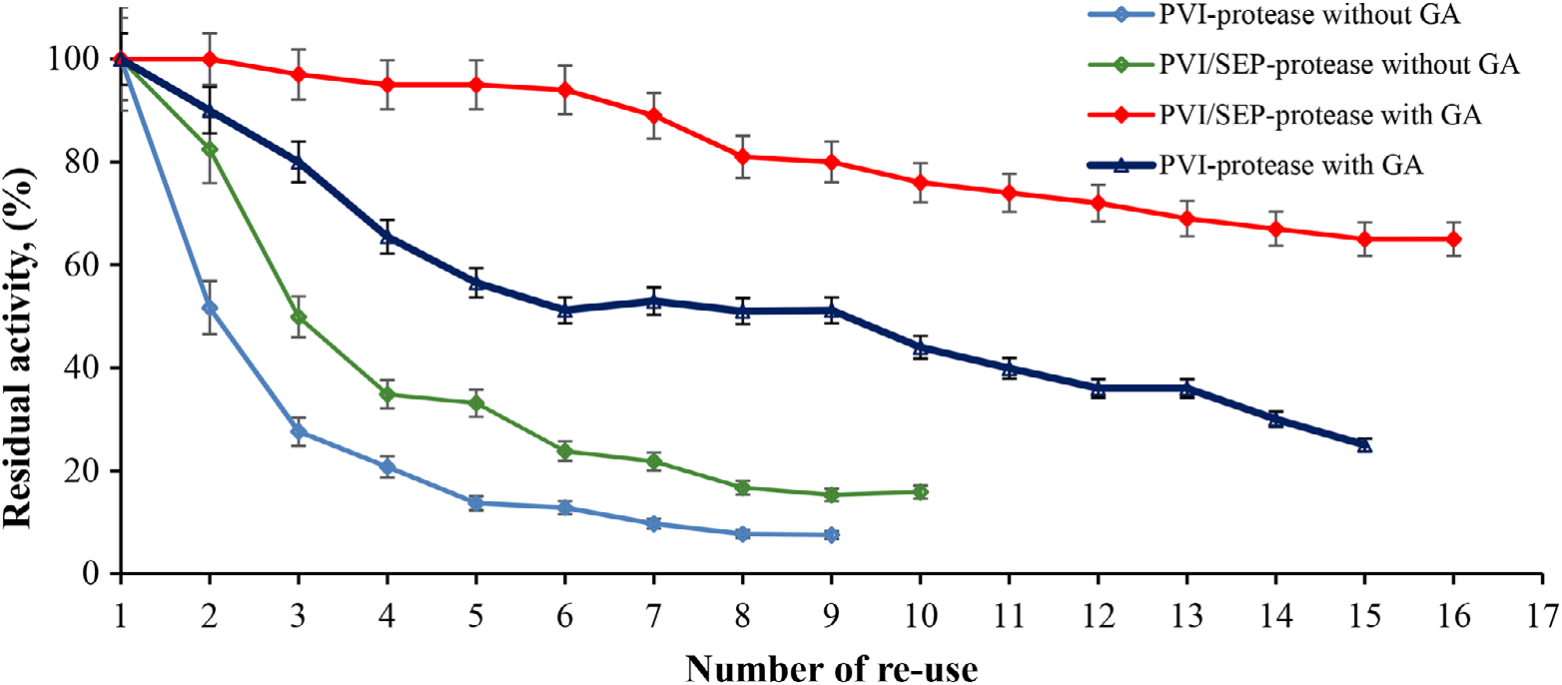
The effects of glutaraldehyde and SEP were determined on the reuse of immobilized enzyme. (◊) Reuse‐ability of PVI‐protease immobilization in the absence of glutaraldehyde, (◊) reuse‐ability of PVI/SEP‐immobilized protease in the absence of glutaraldehyde, (♦) reuse‐ability of PVI / SEP‐protease in the presence of glutaraldehyde, (Δ) reuse‐ability of PVI‐protease immobilization in the presence of glutaraldehyde has been shown

The maximal immobilization capacity was observed with PVI. In these experiments, the starting activity of the enzyme and the mass of the support materials was adjusted to 118.5 U/mL and 0.10 g, respectively, in order to allow direct comparison between the immobilization efficiency and the support material. However the relative activity observed for the enzyme immobilized on different material was different. Percent residual activities obtained after the immobilization process for the immobilized alkaline protease, representing the IE, were 95, 85, and 20%, in the presence of PVI, PVI/SEP, and SEP, respectively. This effect could be attributed to the specific interactions of the enzyme with PVI, SEP, and PVI/SEP 33. Although high immobilization efficiency is important, the reusability of an immobilized enzyme has more importance in industrial applications 34. The reusability of the alkaline protease enzyme immobilized with PVI and PVI/SEP was evaluated in batch operation mode. The reusability of the immobilized alkaline protease through physical adsorption has been presented in Figure 5. The reuse number and the percentage of residual activity of the immobilized alkaline protease were observed to increase in the presence of GA. The PVI/SEP‐immobilized alkaline protease, in the presence of GA, maintained its initial activity even in the sixth cycle of use. Moreover, only 35% of the initial activity was lost at the end of the 16^th^ cycle of use. The increase in the reusability and in the percentage residual activity in the presence of GA may be explained on the basis of enhanced cross‐linking leading to the aggregation of alkaline protease. Gathered alkaline protease due to the effect of the GA enzyme could not escape through the pores of the support material. GA may also lead to smaller pore size and greater entrapment of the alkaline protease molecules 35. In the enzyme immobilization processes, chemical cross‐linking of the enzymes by GA has gained considerable attention recently. Homo‐bifunctional cross‐linkers such as GA contain two identical reactive dialdehyde groups with five carbon atoms. The reaction of these groups with the protein molecules requires just one step, i.e. the intra‐molecular cross‐linking, which may stabilize the protein's quaternary structure. Crude protein molecules exhibit the highest stability in vivo. When these molecules are placed in vitro, most cases will result in denaturation. In comparison to the corresponding soluble protein molecule, chemical cross‐linking of the protein molecules via GA may exceptionally enhance their stability against denaturation or decomposition 36. The immobilized protease was observed to retain 30% of its initial activity and at the end of the 12^th^ cycle of use 37. It has been reported that retention of greater than 60% of the initial activity of the immobilized protease enzyme post an activity cycle indicates successful immobilization 38. Therefore, the present study is supported by this finding as the relative activity observed in the present study was 78%.

### 3.4 Operational stability of immobilized alkaline protease

**Figure 6:**
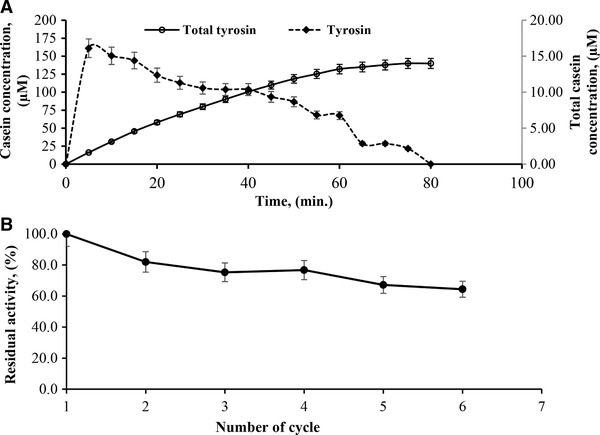
(A) Determination of operational stability of immobilized alkaline protease on PVI/SEP hydrogel as a residual tyrosine concentration (open circle) and total tyrosine concentration (closed quadrangle) in a bulk reaction solution. (B) Operational stability of immobilized alkaline protease on PVI/SEP hydrogel in subsequent six cycles

Since the costs of enzyme production are high, operational stability of the immobilized enzyme serves as an important parameter for determining the enzyme's potential application in the industry. Therefore, immobilization of enzymes must lead to high operational stability. The operational stability of the PVI/SEP‐immobilized alkaline protease was studied batch‐wise, in a flask under mild shaking conditions, with 20 mL of 0.6% casein solution and 0.1 g of the immobilized enzyme (specific activity: 135.4 U/mg). Each cycle continued for 80 min until 95% decrease in the amount of casein at pH 8 was reached. The data related to the operational stability of the immobilized alkaline protease are presented in Figure 6A and B. A 35% loss of activity was observed after six cycles of operation at 30°C in the immobilized alkaline protease. Nayera et al. demonstrated the operational stability of the immobilized enzyme through five rounds of substrate hydrolysis. Conversion decreased in the second round, reaching 35%, after which it remained relatively stable through the subsequent fifth rounds 38. Higher stability could have been due to the specific nature of the alkaline protease derived from *Bacillus pumiluspumilus* Y37.

After 80 min (Figure 6A), the immobilized alkaline protease was recovered through simple decantation. The extent of hydrolysis was determined by testing the supernatant for tyrosine. The recovered immobilized alkaline protease was subsequently used as a catalyst for another reaction mixture containing fresh substrate. This process was repeated for a total of six rounds of hydrolysis (total duration of experiment = 8 h).

Immobilized protease enzyme was stored with 100% activity at 4°C over a period of two weeks. The percentage residual catalytic activity for free protease after 60 days of storage at 4°C was 70%.

### 3.5 Effect of pH and temperature on immobilized enzyme

**Figure 7:**
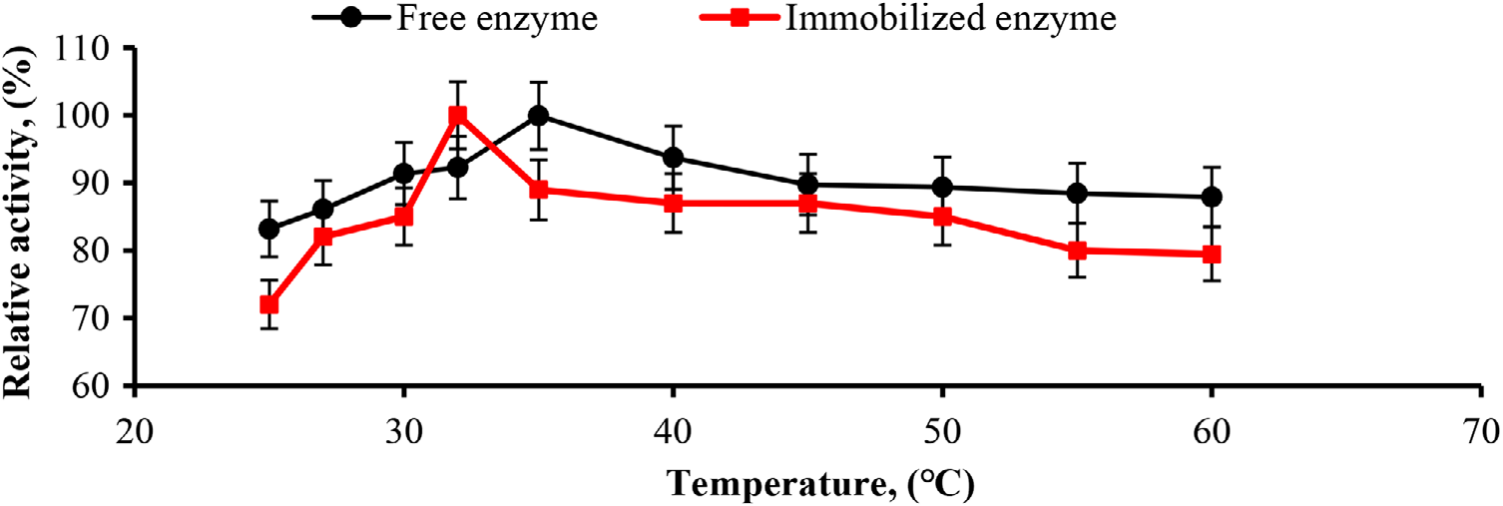
Determination of optimal temperature for immobilized (■) and free enzyme (●). The reaction was carried out by the standard activity determination protocol. All experiments were performed in duplicate, and the results represented the mean values of three independent experiments

**Figure 8:**
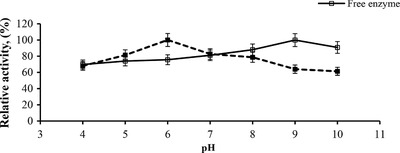
Determination of optimal pH for immobilized (■) and free enzyme (□). The reaction was carried out by the standard activity determination protocol. All experiments were performed in duplicate, and the results represented the mean values of three independent experiments

The temperature activity and stability profile of an enzyme are considered one of the important factors determining its potential for industrial applications. The relationship between the temperature and the activity of the free and immobilized alkaline protease enzymes was evaluated by measuring the initial reaction rate at pH 8.0 at a broad temperature range (25–60°C). The relative activity, denoted as the percentage of the maximum activity, is expressed as a function of temperature in Figure 7. The maximal activities were obtained at 35 and 32°C for the free and the immobilized alkaline protease, respectively (Figure 7). A slight shift was observed in the temperature optimum of the initial reaction rate of the immobilized alkaline protease, in comparison to the temperature optimum of 35°C obtained for the free enzyme. This small change observed in the optimum temperatures of the free and the immobilized enzymes might have been caused by a change in the electrostatic interactions in the conformational integrity of the enzyme upon the immobilization of the enzyme with PVI/SEP hydrogel. The optimum pH for the activity of the free and immobilized alkaline protease enzymes was determined by measuring the initial reaction rate at different pH values (from 4 to 10) at 30°C. As depicted in Figure 8, the maximum activities of the free and immobilized enzymes were obtained at pH 9 and 6, respectively. The shift in the optimal pH value for the PVI/SEP–alkaline protease may have occurred due to stronger interactions between the enzyme and the carrier material, including hydrogen bonding and electrostatic interactions 39, 40. Moreover, it is speculated that the possible aggregations by formations of Schiff base between amino and carboxyl groups of protease caused by GA could have caused the pH shift.

### 3.6 Kinetic parameters of PVI/SEP–alkaline protease

**Figure 9:**
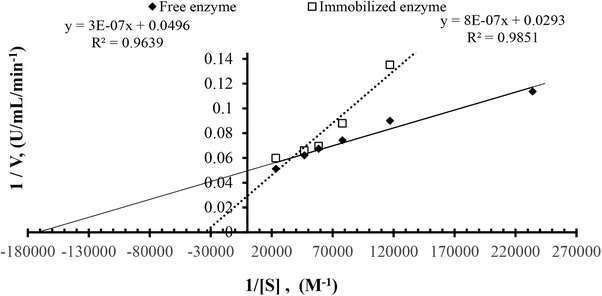
Lineweaver–Burk plot for immobilized (□) and free alkaline protease (♦) with is casein substrate

The kinetic studies of the free and immobilized enzymes were performed using different concentrations of casein. Activity measurements were taken in 50 mM K_2_HPO_4_‐KH_2_PO_4_ buffer (pH 8). The values of maximal velocity (*V*_max_) and the Michaelis–Menten constant (*K*_m_) were calculated from a Lineweaver–Burk plot, and were determined to be 6.05 × 10^−6^ M casein and 20.16 U min^−1^ for the free protease; respectively, for the immobilized alkaline protease, and 27 × 10^−6^ and 34.13 U min^−1^, respectively, for the PVI/SEP–alkaline protease (Figure 9 and Table 2). A five‐fold increase in the *K*_m_ value was observed for the immobilized enzyme compared to the free enzyme; higher *K*_m_ values reflect lower affinity between substrate and enzyme 39. The increased *K*_m_ values obtained for the PVI/SEP–alkaline protease could be attributed to the mass transfer resistance and a decline in the flexibility of the enzyme molecule, thereby causing the substrate to remain in the immobilization matrices, resulting in the reduced ability of the substrate to reach the alkaline protease active site 35, 41. Similar results were reported in previous studies as well, where the *K*_m_ values increased after the immobilization of alkaline protease. The studies conducted on the immobilization of different enzymes on various supports have suggested that the *K*_m_ value of the enzyme is altered as a result of the immobilization (Table 3) 39, 41, 42.

Increasing *V*_m_ value of the PVI/SEP–alkaline protease in comparison to that of the free alkaline protease could be attributed to the increased stability of the enzyme post immobilization. Increase in *K*_m_ and *V*_max_ values post immobilization has been reported in previous studies, and these findings are consistent with the results of the present study (Table 3) 39, 42. The value for *k*_cat_ is calculated by dividing *V*_max_ with the total enzyme concentration. *k*_cat_ of immobilized enzyme was increased compared with native enzyme (Table 2). Although the *K*_m_ value of the immobilized enzyme was increased with diffusion restriction, the multi‐point interaction of the enzyme with PVA/SEP by physical adsorption was significantly increased with the reaction rate (Table 2). This may indicate that the substrate binds to the active site more strongly than the transition form due to minor conformational changes in the enzyme. As previously reported (Table 3) [43,44], this corresponds to enzyme‐substrate complementarity to enzyme‐substrate complex binding energy greater than the state of enzyme transition, resulting in the decrease in both *k*_cat_ and *K*_m_. In our study, to determine the presence or absence of diffusional limitation, biochemical catalysis of N‐Suc‐L‐Ala‐Ala‐Pro‐Phe‐*p*‐nitro aniline ester by *B. pumilus* alkaline protease was also investigated. *K*_m_ and *k*_cat_ values for free and immobilized were 4.02 × 10^−3^ M and 120.4 min^−1^ and 4.84 M and 128.6 min^−1^, respectively. These results can be expressed as diffusional limitation for casein substrate which is too large relative to the ester of N‐Suc‐L‐Ala‐Ala‐Pro‐Phe‐*p*‐nitro aniline ester.

### 3.7 Activation energy of immobilized enzyme

**Figure 10:**
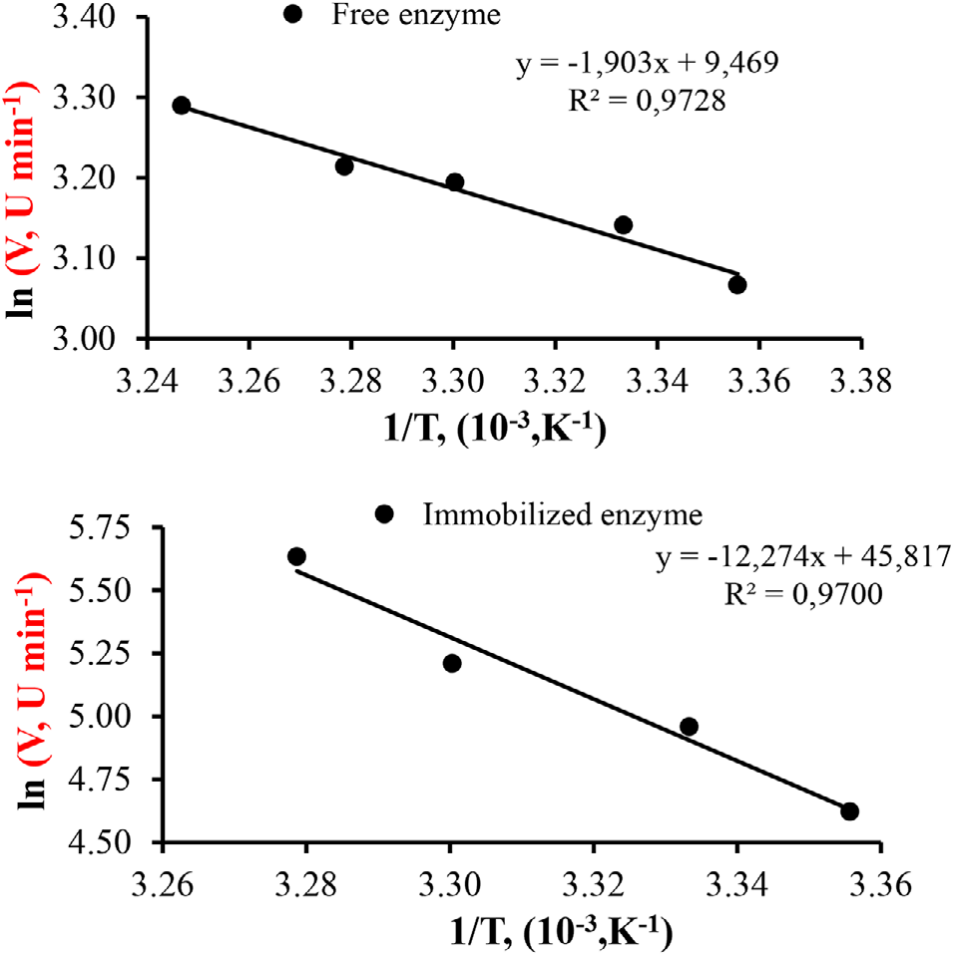
Arrhenius plot for the identification of activation energy of free and immobilized alkaline protease

The activation energies of the free and PVI/SEP‐immobilized alkaline protease enzyme were determined as 15.82 and 102 kJ/mole, respectively (Figure 10). Although it has been reported that multi‐point interactions of the alkaline protease molecule with the support might cause a decrease in the conformational flexibility of the enzyme, thereby resulting in higher activation energy 38, there are also studies available in the literature that report an increase in the conformational flexibility of the enzyme 9.

### 3.8 Thermodynamic parameters of the immobilized enzyme

Thermodynamic parameters of the free and immobilized alkaline protease enzyme indicated that the values of ΔG^≠^ (activation free energy) and ΔG^≠^_E‐T_ (activation free energy of transition state formation) obtained for the immobilized enzyme decreased in comparison to those obtained for the free enzyme. On the other hand, the value of ΔG^≠^_ES_ (free energy of substrate binding) was observed to have increased (Table 1).

**Table 1:** Purification profile of the *Bacillus pumilus* Y7 alkaline protease

Step	Volume (mL)	Total protein, (mg)	Total activity, (U)	Spesificactivity, (U/mg)	Purificationfold	Yield, (%)
Homogenate	950	3900	48 500	12.43	1	100
Phenyl‐Sepharose‐6 fastflow	250	372.47	25 600	67.12	5.5	52.8

**Table 2:** Kinetic and thermodynamic properties of immobilized and free alkaline protease

Parameter	Free protease	PVI/SEP‐protease	Unit
*k*_cat_	24.39 ± 1.24	180.4 ± 12.43	min^−1^
*K*_m_	6.05 × 10^−6^ ± 3 × 10^−7^	27 × 10^−6^ ± 7 × 10^−7^	M
*V*_m_	20.16 ± 5.44	34.13 ± 12.24	U mL^−1^ min^−1^
*E*_a_	15.82 ± 3.74	102.04 ± 12.21	kJ mole^−1^
∆G^#^	66.21 ± 9.32	61.15 ± 8.41	kJ mole^−1^
∆H^#^	13.3 ± 2.29	29.52 ± 6.24	kJ mole^−1^
∆S^#^	−0.17 ± 0.01	0.12 ± 0.02	kJ mole^−1^K^−1^
∆G^#^_E‐T_	−38.31 ± 12.56	−56.22 ± 14.24	kJ mole^−1^
∆G _E‐S_	−30.27 ± 11.13	−26.45 ± 9.38	kJ mole^−1^

**Table 3:** Comparison of catalytic properties of an enzyme and its immobilized form

Parameter	Free enzyme	Immobilized enzyme	Ref.
*k*_cat_	283 s^−1^	333s^−1^	42
*K*_m_	2.5 mM	3.8 mM	39
	0.87 mg/mL	2.6 mg/mL	40
*V*_m_	3.02 U/mg protein	3.35 U/mg/protein	39
	0.72 mg/mL/min	2.0 mg/mL/min	40
*E*_a_	1.87 kcal/mol K	4.69 kcal/mol K	43

Calculation of the changes in ΔG^≠^ for substrate hydrolysis indicated the realization possibility of an enzymatic reaction, especially the conversion of a transition state complex (ES^≠^) into the product. Lower ΔG^≠^ value obtained for the immobilized alkaline protease enzyme implied that the conversion of its transition complex into products was more spontaneous in comparison to that in case of the free enzyme. The substrate binding free energy (ΔG^≠^_ES_) reaches its highest value when all the binding groups on the substrate molecule match the binding site of the enzyme (enzyme–substrate complementarity). However, what is more beneficial to the spontaneity of the biochemical reaction is the complementarity between the enzyme and the transition state form substrate (S^≠^). In such cases, due to the conformational change in the substrate molecule, the free energy of substrate binding increases, while the activation free energy of the reaction decreases. If the substrate in its original conformation is complementary to the enzyme molecule, the free energy of transition state binding (ΔG^≠^_E−T_) decreases, while the activation free energy increases 45. The latter case is completely compatible with the results of the present study, where the ΔG^≠^_ES_ value of the enzyme was observed to increase, while the ΔG^≠^_E−T_ and ΔG^≠^ values of the immobilized alkaline protease enzyme decreased, indicating an increase in the spontaneity of the biochemical reaction post immobilization. On the other hand, the value of ΔH is related to the energy that is necessary for the formation of the transition state. The lower ΔH value obtained for the immobilized enzyme in the present study in comparison to that for the free enzyme implied lower energy for the formation of the transition state, and thus, a convenient product formation. The ordered structure of an enzyme is identified through entropy. In general, the lower entropy (ΔS) obtained for the PVI/SEP‐immobilized alkaline protease enzyme indicated that the immobilized form of the enzyme was more ordered than its free form.

## 4 CONCLUDING REMARKS

The present study investigated the purification and immobilization of *Bacillus pumilus* Y37‐derived alkaline protease enzyme with PVI/SEP hydrogel. As a result of this immobilization, both activation energy of the reaction and the catalytic performance of the enzyme were observed to increase. One of the most important parameters in the enzyme immobilization is the reusability of the enzyme. In the present study, the activity of the enzyme was almost completely maintained until seven activity cycles. In the 16^th^ cycle, 65% of the initial activity of the enzyme was observed to have been retained. In the present study, the efficiency obtained after the immobilization of the enzyme with PVI/SEP was 85%. PVI/SEP is suitable support material for the immobilization of enzymes, with a potential for industrial applications as it provides high efficiency, reusability of the enzyme, and high catalytic performance. PVI/SEP may also be a preferred choice for support material for the immobilization of enzymes because of its biocompatibility, convenient synthesis, and moderate cost. According to the findings of the present study, PVI/SEP is suitable for use as a support material for enzyme immobilization in the industries.

## CONFLICT OF INTEREST

The authors have declared no conflict of interest.
